# Co-existence of Blau syndrome and NAID? Diagnostic challenges associated with presence of multiple pathogenic variants in NOD2 gene: a case report

**DOI:** 10.1186/s12969-017-0188-7

**Published:** 2017-07-27

**Authors:** Magdalena Dziedzic, Agata Marjańska, Katarzyna Bąbol-Pokora, Anna Urbańczyk, Elżbieta Grześk, Wojciech Młynarski, Sylwia Kołtan

**Affiliations:** 1 Department of Paediatrics, Haematology and Oncology Nicolaus Copernicus University in Toruń, Collegium Medicum in Bydgoszcz, Bydgoszcz, Poland; 2 Department of Paediatrics, Oncology, Haematology and Diabetology Medical University of Łódź, Łódź, Poland; 3 Department of Paediatrics, Haematology and Oncology, Antoni Jurasz University Hospital, No. 1 in Bydgoszcz, ul. Skłodowskiej-Curie 9, 85-094 Bydgoszcz, Poland

**Keywords:** Blau syndrome, NAID, Autoinflammatory disease

## Abstract

**Background:**

Pediatric autoinflammatory diseases are rare and still poorly understood conditions resulting from defective genetic control of innate immune system, inter alia from anomalies of NOD2 gene. The product of this gene is Nod2 protein, taking part in maintenance of immune homeostasis. Clinical form of resultant autoinflammatory condition depends on NOD2 genotype; usually patients with NOD2 defects present with Blau syndrome, NOD2-associated autoinflammatory disease (NAID) or Crohn’s disease.

**Case presentation:**

We present the case of a 7-year-old girl with co-existing symptoms of two rare diseases, Blau syndrome and NAID. Overlapping manifestations of two syndromes raised a significant diagnostic challenge, until next-generation molecular test (NGS) identified presence of three pathogenic variants of NOD2 gene: P268S, IVS8_+158_, 1007 fs, and established the ultimate diagnosis.

**Conclusion:**

Presence of multiple genetical abnormalities resulted in an ambiguous clinical presentation with overlapping symptoms of Blau syndrome and NAID. Final diagnosis of autoinflammatory disease opened new therapeutic possibilities, including the use of biological treatments.

## Background

Pediatric autoinflammatory diseases are rare and still poorly understood conditions characterized by presence of inflammatory reaction despite the lack of exposure to an extrinsic infectious agent and resultant response of antigen-specific T cells. Autoinflammatory conditions result from defective genetic control of innate immune system [1]. One of affected genes can be NOD2 located on the long arm of chromosome 16 (16q12). The product of this gene is Nod2 peptide (CARD15) from the family of leucine-rich repeat receptors. A balanced level of NOD signaling is essential for the maintenance of immune homeostasis [2]. Upregulation of Nod2 is typical for antigen-presenting cells (APC), such as monocytes, macrophages and intestinal Paneth cells [3]. Through peptidoglycan recognition, the nucleotide-binding oligomerization domain (NOD) proteins NOD1 and NOD2 enable detection of intracellular bacteria and promote their clearance, activating a pro-inflammatory transcriptional program and other host defense pathways, including autophagy [2]. A total of 144 variants of NOD2 gene have been described thus far, along with their specific phenotypes. Clinical form of resultant autoinflammatory condition depends on NOD2 genotype; usually patients with NOD2 defects present with Blau syndrome, NOD2-associated autoinflammatory disease (NAID) or Crohn’s disease [4–6]. However, some patients, especially those with several co-existing abnormalities in NOD2 gene, do not satisfy diagnostic criteria of any of the abovementioned conditions, or present with overlapping symptoms of more than one autoinflammatory disease.

## Case Presentation

We present the case of a 7-year-old Caucasian girl, from the second gestation and parturition, a daughter of healthy unrelated parents. The child was born in a good general condition, with birth weight of 3780 g and 51-cm body length. Gestational, perinatal and family histories were unremarkable.

**Fig. 1:**
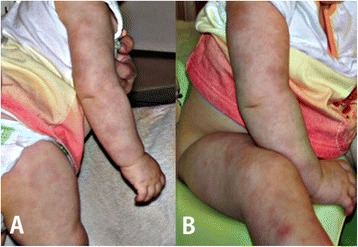
A 12-month-old patient with eczematous dermatitis. Photographs A and B show erythematous maculo-micropapular skin lesions with some changes in type of livedo reticularis

At the age of 2 years, the patient has been hospitalized for the first time in our clinic due to anemia, leucopenia, hepatosplenomegaly and more than a 6-month history of recurrent lymphadenopathy. Moreover, the girl had a history of a non-specific whole-body intermittent skin rash (Fig. 1) and recurrent fever (<39 °C), both starting at 4 months of age and recurring at irregular intervals. The presence of skin lesions was independent of the fever. Erythematous maculo-micropapular lesions appeared in variable locations and persisted up to several weeks. Moreover, the patient periodically suffered from arthralgia with inability to move, accompanying swelling and increased warmth of affected joints.

Physical examination revealed elevated body temperature (up to 38 °C), skin pallor, livedo reticularis, eczematous dermatitis, multiple enlarged peripheral lymph nodes, persistent front fontanelle (2 cm × 3 cm), systolic murmur (3/6 in Levine scale) and hepatosplenomegaly. Moreover, the patient presented with pruritic erythematous plaques, macules and linear scratch-like rash on the face, chest, abdomen and limbs (Table 1).

**Table 1:** Characteristics of Blau syndrome and NAID

CHARACTERISTICS			Blau Syndrome	NAID	Patient
Gender		Female > Male	+	+	√
Ethnicity		Caucasian	+	+	√
Age at onset		> 40 years	–	+	–
		< 5 years	+	–	√
Clinical features	Frequent	Uveitis	+++	–	√
		Arthritis / arthralgia	+++	++	√
		Skin rash / dermatitis	+++	+++	√
		Recurrent fever	++	+++	√
		Periodic occurrence	++	+++	√
	Infrequent	Gastrointestinal involvement	–	++	√
		Serositis	–	++	–
		Sicca-like symptoms	–	++	–
		Adenopathy	++	–	√
		Camptodactyly	++	–	–
		Malignant hypertension	++	–	–
		Lung involvement	++	–	–
		Kidney involvement	++	–	–
		Hepatosplenomegaly	++	–	√
		Neurological symptoms	++	–	√
		Vasculitis	++	–	–
Gene mutations		NOD2: R334W	+++	–	–
		NOD2: R334Q	+++	–	–
		NOD2: P268S	+	–	√
		NOD2: IVS8_+158_	–	+++	√
		NOD2: R702W	–	++	–
		NOD2: G908R	–	++	–
		NOD2: 1007 fs	–	++	√
Laboratory data		Leukocytosis	++	++	–
		Anemia	++	++	√
		Elevated acute phase reactants	++	++	√
		Presence of antinuclear antibodies	++	+	–
		Elevated Il-1β, Il-6, TNF	++	++	–
		Low total IgG or/and IgM/IgA levels	+	++	√
Other tests	Skin biopsy	Granulomatous dermatitis	+	–	√
		Spongiotic dermatitis	–	+	–
	Endoscopy	Inflammatory bowel disease	–	–	–

Legend: “+++“ - characteristic; “++“ - common; “+“ - rare; “–“ – non-characteristic

A few potential diagnoses were considered on the differential, among them an infection, metabolic or autoimmune disease, proliferative processes and immunodeficiency syndrome. Extensive laboratory workup revealed leukopenia with lymphopenia (WBC = 2.41 × 10^9^/l; LYMPH = 0.74 × 10^9^/l), without accompanying disorders of lymphocyte subpopulations, microcytic anemia (HB = 8.2 g/dl; MCV = 59.4 fl), anisocytosis and anisochromia on manual blood smear, slightly elevated level of C-reactive protein (9.28 mg/l), lowered concentration of iron without other iron irregularities, deficiency of class M and G3 immunoglobulins, and vitamin D3 deficiency. Abdominal ultrasound scan (USS) documented enlargement of the liver (30 mm below the right costal arch) and spleen (length 118 mm, with age-specific reference range up to 95 mm), with normal echogenicity of both organs. Peripheral lymph node USS revealed presence of suspicious (hypoechogenic, up to 20 mm in diameter, without a marked sinus) supraclavicular, infraclavicular and axillary lymph nodes. Histopathological examination of one of the left supraclavicular lymph nodes showed reactive changes with neutrophilic infiltration. Examination of skin biopsy specimen demonstrated presence of multiple granulomas composed of giant cells and epithelial cells at the border of the skin and subcutaneous tissue, raising a suspicion of sarcoidosis or erythema annulare. However, further diagnostic process was interrupted since the patient and her family went abroad for a few years. Medical documentation from this period, provided by a foreign clinic, is incomplete. Apparently, the patient was diagnosed with a sporadic sarcoidosis, and treated with methotrexate and prednisone. However, the therapy did not produce a satisfactory outcome; furthermore, during therapy the patient developed anterior uveitis and hydrocephalus that required shunting.

**Fig. 2:**
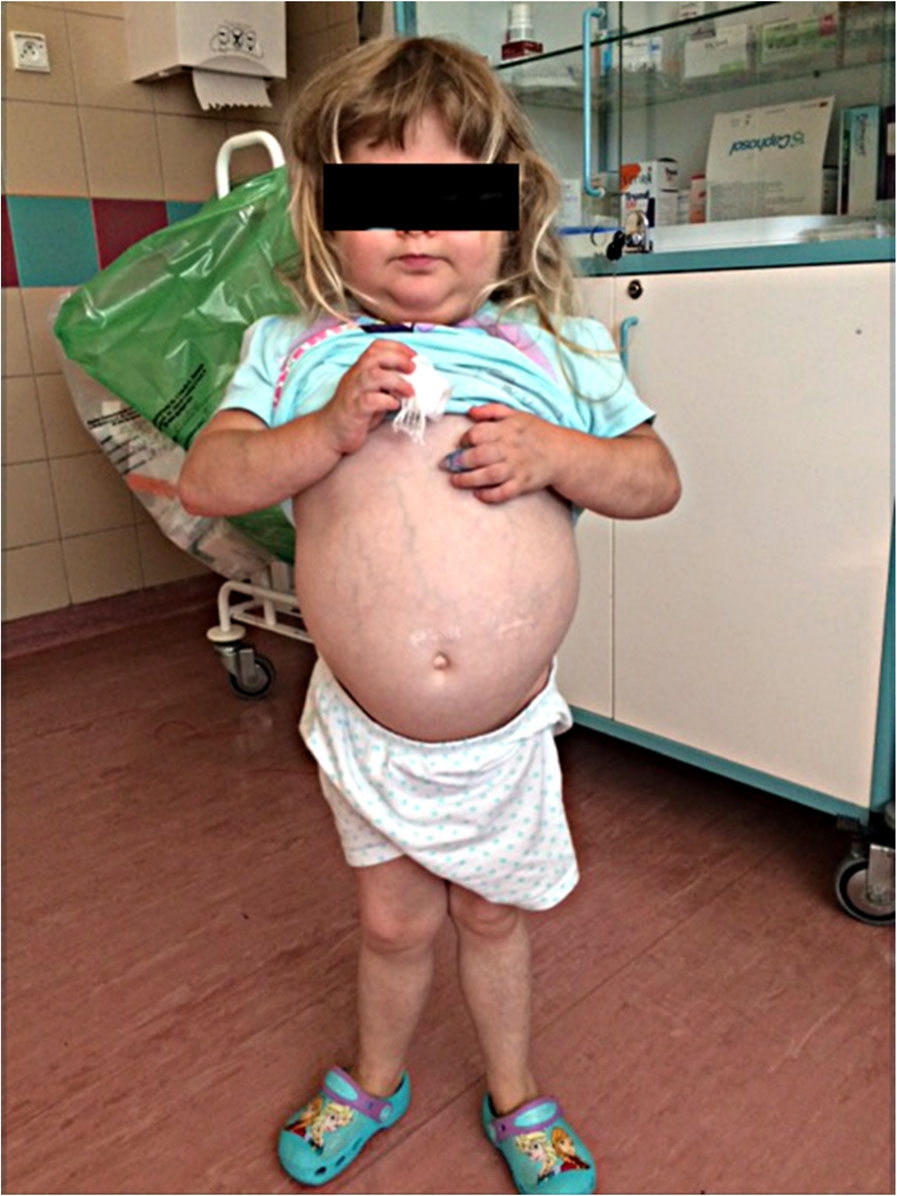
A 7-year-old patient after prolonged corticosteroid therapy. Note some physical features of iatrogenic Cushing syndrome: moon facies, facial plethora, truncal obesity, thin arms and legs, purple striae

Four years later, the patient was readmitted to our clinic. Her general condition has deteriorated, her mobility was decreased, and she presented with severe symmetrical knee and ankle arthritis without erosive and destructive changes in the joints. Tenosynovitis and synovitis were excluded. While the severity of eczematous dermatitis decreased, multiple subcutaneous nodules emerged on both upper and lower limbs, as well as on the torso; the skin lesions responded well to escalation of glucocorticoid dose. The intervals with normal body temperature were shorter than before, and any attempt to reduce the glucocorticoid dose resulted in recurrence of the fever. Furthermore, the patient presented with recurrent diarrhea and concomitant abdominal pain. Other abnormalities found on physical examination included Cushinoid physique, left eye amblyopia persisted after the episode of uveitis, neurological deficits, non-active hydrocephalus, non-neuromuscular weakness in the upper and lower limbs, short stature (<3 pc) and genu valgum (Fig. 2). While neurological and psychomotor development of the patient was normal before she reached 2 years of age, a plethora of neurological disorders emerged when she stayed abroad, following manifestation of hydrocephalus. Laboratory workup documented further progression of anemia, leukopenia and lymphopenia, extremely low concentration of main class immunoglobulins, inability to produce specific antibodies, and elevated percentage of CD3 + TCRγδ + CD4-CD8- cells. Also a slight although progressive increase in inflammatory marker levels was demonstrated. Due to presence of gastrointestinal disorders, the patient was subjected to endoscopic examination which, however, revealed no abnormalities. The evidence from brain MRI suggested the presence of chronic meningoencephalitis.

The results of the abovementioned tests raised a suspicion of common variable immunodeficiency (CVID) or an autoinflammatory syndrome with secondary immunodeficiency resulting from prolonged methotrexate and corticosteroid therapy.

As the symptoms were not controlled adequately with previous treatments, cyclosporine (3 mg/kg/day) and intravenous human immunoglobulins at a supplementation dose (0.4 g/kg/month) were added to the therapeutic protocol. Systemic corticosteroid therapy was continued with the intention to gradually taper down the dose. Modification of therapeutic protocol resulted in a partial response: reduction of body temperature, improvement of mobility, attenuation of arthralgia and neurological symptoms, and nearly complete regression of the changes found previously on brain MRI. However, an evident steroid dependence was observed, as each attempt to taper down the dose resulted in the recurrence of the symptoms. Furthermore, the features of iatrogenic Cushing’s syndrome exacerbated. Taken all together, this clinical presentation suggested that biological treatment should be taken into consideration.

Owing ambiguous clinical presentation and high likelihood of a primary immunodeficiency syndrome, the patient was qualified for molecular testing with next-generation sequencing (NGS) method. The tests identified three heterozygous anomalies in NOD2 gene: two substitutions, including one within the 8th intron (2798 + 158C > T; 802C > T;), and one insertion (3016_3017insC). 802C > T is a proline to serine substitution in locus 268. Insertion of cytosine (3016_3017insC) between nucleotide 3016 and 3017 of NOD2 gene results in a frameshift change causing premature termination of the protein chain (Leu1007ProfsTer2). Based on the abovementioned findings, the patient was diagnosed with NOD2-associated autoinflammatory disease, which justified switching her to a biological treatment. Patient’s parents decided to continue the treatment in a clinic abroad, whereby adalimumab therapy was implemented. Corticosteroids have not been completely discontinued, but tapered down to 1 mg/m^2^/day, which resulted in a gradual regression of iatrogenic Cushing syndrome. Patient’s quality of life improved, she began attending school, but still needs a psychological support and physiotherapy. Also, immunoglobulin supplementation is continued at 4-week intervals. Finally, genetic counseling was recommended to the patient’s parents. Both parents tested negatively for mutations in NOD2 gene.

## Discussion

We present the case of a 7-year-old girl with co-existing symptoms of two rare diseases. Clinical characteristics of Blau syndrome and NAID are listed in Table 1, along with the signs and symptoms present in our patient [1, 3, 5, 7–10]. As shown in the table, clinical phenotype of our patient with overlapping manifestations of NAID and Blau syndrome, correlated with her genotype; molecular tests demonstrated that our patient carried two alterations characteristic for NAID (1007 fs, IVS8_+158_), as well as a genetic anomaly that had been previously found in a child with Blau syndrome (P268S) [5, 7, 10]. Early-onset sarcoidosis (EOS) was also considered during the differential, although in line with currently used classification system, this condition represents a sporadic form of Blau syndrome [11, 12]. Principal diagnostic criteria of NAID include recurrent, self-limiting episodes of inflammation lasting days to weeks, presence of fever of unknown etiology and/or dermatitis [7]. While our patient satisfied all these criteria, she was much younger than previously reported subjects with NAID. To the best of our knowledge, to this date, NAID has not been reported in a pediatric patient; according to literature, the youngest patient with NAID was aged 17 years, and this condition is typically diagnosed at 40–45 years of age [1, 7–9]. However, early onset is typical for another autoinflammatory disease, Blau syndrome. Indeed, some symptoms present in our patient (uveitis, granulomatous dermatitis, hepatosplenomegaly, neurological manifestations, adenopathy were not typical for NAID, but have been previously reported during the course of Blau syndrome.

Two most common variants of NOD2 gene found in patients with NAID are 2798 + 158C > T (IVS8^+158^) and R702W [7, 9]. We found the former one in our patient, along with another two NOD2 variants: Leu1007ProfsTer2, also documented in individuals with NAID [4, 5], and P268S that has been previously reported in a child with typical clinical presentation of Blau syndrome. Furthermore, all three genetic variants found in our patient may predispose to Crohn’s disease; however, no clinical evidence of the latter condition has been found thus far. Nevertheless, it cannot be excluded that the patient may develop Crohn’s disease later in life, and consequently she should be regularly checked for potential manifestations of this condition. Furthermore, Leu1007ProfsTer2, one of the genetic variants of NOD2 identified in the hereby presented case, was shown to be associated with other clinical entities, such as psoriatic arthritis, rheumatoid arthritis, spondyloarthropathy and ulcerative colitis [6].

Although some patients with NAID may present with hypogammaglobulinemia, severe immunoglobulin deficiency is not a typical feature of this condition. However, if developed, this rare manifestation may occasionally require administration of human immunoglobulins. Diagnostic criteria of CVID are still being modified. The last modification, developed by the ESID, dates back to 2014, and includes clinical and laboratory criteria, among them a decrease in IgG and IgA, with or without a concomitant reduction of IgM level [13]. In our patient, symptoms raising a suspicion of an autoinflammatory syndrome (CVID typically manifests with autoimmune symptoms) appeared in the first year of life, and IgM deficiency was first observed during the initial hospitalization at 2 years of age, followed by a decrease in concentrations of immunoglobulins from all classes during the second hospital stay, when dual immunosuppression therapy was administered.

CVID is usually diagnosed in children older than 4 years, and thus, early onset of clinical symptoms virtually excluded this diagnosis in our patient. Further, diagnosis of CVID requires exclusion of secondary hypogammaglobulinemia, and our patient presented with a severe immunodeficiency due to administration of corticosteroids and methotrexate [13].

The diagnostic delay in the presented case (ultimate diagnosis was established no earlier than after 4 years) was caused by a few factors: non-specific symptoms that could not be matched with any known clinical syndrome, treatment at two centers from two different countries, and last but not least, unavailability of genetic testing with NGS at early stages of the disease. Positive result of molecular testing opens new therapeutic perspectives, and justifies implementation of biological treatments with monoclonal anti-TNF antibodies (infliximab, adalimumab, tocilizumab) or anti-IL-1B antibodies (canakinumab) [5, 7, 14].

## Conclusions

In this case report, we describe a child with co-existing symptoms of two rare diseases, Blau syndrome and NAID. Overlapping symptoms of two syndromes raised a significant diagnostic challenge, until next-generation molecular test (NGS) identified presence of three pathogenic variants of NOD2 gene, explaining concomitant manifestation of Blau syndrome and NAID. Final diagnosis of the autoinflammatory syndrome opened new therapeutic perspectives, including implementation of a biological treatment.

## Abbreviations

APC: Antigen-presenting cells
CVID: Common variable immunodeficiency
EOS: Early-onset sarcoidosis
HB: Hemoglobin
LIMF: Lymphocytes
MCV: Mean cell volume
MRI: Magnetic resonance imaging
NAID: NOD2-associated autoinflammatory disease
USS: Ultrasound scan
WBC: White blood cells
